# Sucrose and KF quenching system for solution phase parallel synthesis

**DOI:** 10.1186/s40064-016-2394-z

**Published:** 2016-07-12

**Authors:** Sunil Chavan, Rahul Watpade, Raghunath Toche

**Affiliations:** Department of Chemistry, K. T. H. M. College (Affiliated to Savitribai Phule Pune University), Gangapur Road, Nashik, Pune, 422002 India

**Keywords:** KF, Sucrose, Acid chlorides, Sulfonyl chlorides, Isocyanates, Parallel synthesis

## Abstract

The KF, sucrose (table sugar) exploited as quenching system in solution phase parallel synthesis. Excess of electrophiles were covalently trapped with hydroxyl functionality of sucrose and due to polar nature of sucrose derivative was solubilize in water. Potassium fluoride used to convert various excess electrophilic reagents such as acid chlorides, sulfonyl chlorides, isocyanates to corresponding fluorides, which are less susceptible for hydrolysis and subsequently sucrose traps these fluorides and dissolves them in water thus removing them from reaction mixture. Various excess electrophilic reagents such as acid chlorides, sulfonyl chlorides, and isocyanates were quenched successfully to give pure products in excellent yields.

## Background

The generation and use of combinatorial chemical libraries for the identification of novel chemical leads or for optimization of a promising lead candidate has emerged as a potentially powerful tool for acceleration of the drug discovery process (Terrett et al. 1995; Gallop et al. 1994; Gordon et al. 1994; Janda 1994; Pavia et al. 1993). The combinatorial chemistry has already yielded several compounds that are currently undergoing clinical trials. The pharmaceutical industry needs large and diverse molecular libraries. The screening of large number of compounds can be quickly lead to early structure-activity relationships (SARs), and may provide a practical starting point for drug discovery program, where little or no information is known about the target.

**Fig. 1:**
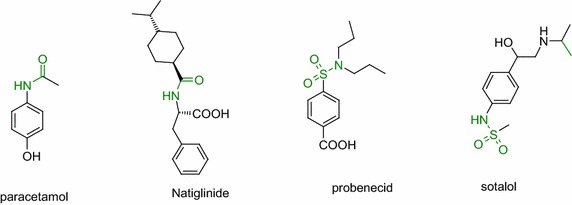
Pharmaceutically active compounds containing amide, sulphone amide groups

The amide and sulfonamide functionalities are the key structural moieties in many pharmaceutically active compounds such as paracetamol (analgesic and antipyretic), nateglinide (for treatment of type 2 diabetes), probenecid (uricosuric drug), and sotalol (for cardiac arrhythmias) (Fig. 1). Similarly, urea functionality act as a non hydrolysable surrogate of amide bonds in many pharmaceutically active molecules (Majer and Randad 1994; Kruijtzer et al. 1997; Decieco et al. 1997). Therefore practical method for rapid synthesis of amide, sulfonamide and urea containing molecules are of great interest in drug discovery and lead optimization.

Solution-phase parallel synthesis is an excellent way to form libraries of small molecules containing amide, sulfonamide and urea functionalities. However, during the synthesis of library, the chemistry for solution phase parallel synthesis require complete conversion of reactants with little or no formation of by-products or impurities to simplify the tedious purification processes. The solid phase synthesis offers benefit of easy and fast purification to separate excess reagents and side products from the desired compounds attached to the insoluble carrier. However, due to heterogeneous reaction conditions, salvation of bound species, and mass transfer of reagents are the limitations of solid phase synthesis. The range of chemistry applied to the solid-phase synthesis has limitations.

In earlier reports, the solid phase quenchers in the form of reagents on the solid phase or ion exchange resins have been used in quenching reactions to eliminate the reactive entites (Thompson and Ellman 1996). Potassium sarcosinate and fluorous-tethered as quencher were also reported to achieve final compounds in good purity and quantities (Nikam et al. 1998; Lindsley et al. 2002; Curran 2001; Zhang et al. 2003; Zhang et al. 2002). Here, we report sucrose as readily available, environment friendly and cost effective novel quenching agent for the solution phase parallel synthesis.

## Results and discussion

**Fig. 2:**
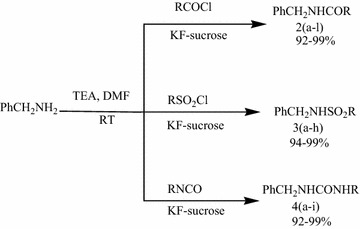
Synthesis of amide **2**; sulfonamide **3** and urea derivatives **4**, reaction mixture was room temperature for 5–6 h

In medicinal chemistry program we need rapid synthesis of various acylated and sulfonated derivatives of benzyl amines. During the parallel synthesis, for the complete conversion of the substrate a little excess of an electrophilic reagent was always added, this resulted the impure products and required tedious column chromatographic purifications. Therefore, a new and rapid process for eliminating these excess electrophiles is required. To overcome this issue, KF and sucrose treatment was found to be a novel quenching system in these reactions. As a prototype we chose benzylamine as substrate (Fig. 2). Thus, in a typical reaction of benzyl amine (1.0 equivalent) was treated with an excess of electrophile 1.4 equivalents, e.g. acid chloride, sulfonyl chloride, or isocyanate in presence of triethylamine in DMF. After stirring for 6 h, potassium fluoride (1.5 equivalents) was added to the reaction and the reaction mixture was stirred for an additional 0.5 h. Further sucrose (1.5 equivalents) was added and reaction mixture stirred for additional 0.5 h. Water was then added to the reaction mixture under stirring and the final product was obtained by simple filtration or extraction with EtOAc. The reaction and the purity of the product were monitored by TLC and ^1^H-NMR, which indicated pure product with no evidence of electrophile used. The products were isolated in excellent yields with high purity. The excess of acid chlorides can also be quenched using aqueous Sodium carbonate, while few aromatic acid chlorides do not get quenched in aqueous base and remain intact; but can be effectively quenched by KF sucrose quenching system (Table 1).

**Table 1:** Isolated yields of amide **2**, sulfonamide **3** and urea **4** derivatives

Amide **2**	R	Yield (%)	Sulfonamide **3**	R	Yield (%)	Urea **4**	R	Yield (%)
a	Cyclopentyl (Sudrik et al. 2002)	98	a	Phenyl (José et al. 2011)	96	a	3-Cyano phenyl	98
b	t-Butyl (Martínez-Asencio et al. 2011a)	99	b	p-Tolyl (Gao et al. 2005)	99	b	1-Naphthyl	99
c	4-Fluro-phenyl (Mamat et al. 2011)	99	c	4-Fluro-phenyl (Ramunno et al. 2012)	97	c	4-Fluro phenyl	97
d	Cyclopropyl (Yang and Shi 2005)	94	d	4-Chlorophenyl (Ramunno et al. 2012)	98	d	2-Trifluromethyl-phenyl	98
e	Cyclohexyl (Saito et al. 2008)	98	e	4-Trifluromethoxy phenyl (Shi et al. 2009)	94	e	2-Methoxy-phenyl	97
f	n-Butyl (Dermer and King 1943)	97	f	4-Methoxy phenyl (Martínez-Asencio et al. 2011b)	99	f	Phenyl	99
g	2-Furyl (Gernigon et al. 2012)	99	g	2,4-Dichloro phenyl	98	g	4-Cyano phenyl	92
h	4-Nitro phenyl (Bahrami et al. 2010)	92	h	3,4-Dichloro phenyl	97	h	4-Triflurometh -oxy phenyl	94
i	4-Methoxy-phenyl (Kokare et al. 2007)	99				i	2,5-Dimethoxyphenyl	99
j	2,4-Dichloro-phenyl (Richard Cremlyna et al. 1989)	95						
k	Ethyl (Lee et al. 1999)	97						
l	Phenyl (Nowrouzi and Jonaghani 2012)	99						

The excess of electrophiles were covalently trapped with hydroxyl functionality of sucrose and dissolve in water. Role of potassium fluoride is to convert various excess electrophilic reagents such as acid chlorides, sulfonyl chlorides, isocyanates to corresponding fluorides (Ishikawa et al. 1981; Kimura and Suzuki 1989; Dang and Olofson 1990), which are less reactive than acid chlorides. Acid fluorides, on the other hand, are known to be more stable to hydrolysis than acid chlorides and hence avoid the formation of by-products. KF is readily soluble in water (92 g/100 ml at 18 °C, 102 g/100 ml at 25 °C) and the reaction in absence of KF yields impure product, which further required column purification. The role of KF is to convert chlorides to corresponding fluorides making them less susceptible to hydrolysis and generation of difficult-to-remove impurities and subsequently sucrose traps these fluorides and dissolves them in water thus removing them from reaction mixture.

After the aqueous quench no sucrose or its derivatives were seen on TLC or in ^1^H NMR of the products. This methodology worked with a larger excess of electrophiles in the reaction, which needed to be quenched with excess amounts of potassium fluoride and sucrose. This methodology can be easily automated on a synthesizer for synthesizing the desire array of compounds in several gram quantities of acylated products.

## Conclusion

In summary, we have developed a simple solution-phase parallel synthesis using a novel quencher for the excess electrophiles. The quencher system KF, sucrose is readily available, environment friendly, cost effective and was found to be very efficient in trapping excess acid chlorides, sulfonyl chlorides and isocyanates to give water soluble by products which could be removed by an aqueous workup.

## Methods

### Reagents used

Reactions were monitored by thin layer chromatography (TLC), carried out on 0.2 mm silica gel 60 F_254_ (Merck) plates using UV light (254 and 366 nm) for detection and compounds were purified by column chromatography by using silica gel of 5–20 µm (Merck, 60–120 mesh). Column dimension 39 × 2 cm and elution volume used is about 200–400 ml for each product. Common reagent grade chemicals are either commercially available and were used without further purification or were prepared by standard literature procedures.

### Characterization

The ^1^H spectra were recorded on a Bruker XL 300 spectrometer (300 MHz). Chemical shifts were reported in parts per million using tetramethylsilane as an internal standard and were given in δ units. The solvent for NMR spectra was DMSO-*d*_*6*_ unless otherwise stated. Elemental analyses were performed on a Hosli CH-Analyzer and are within ±0.4 of the theoretical percentage. All reactions were monitored by thin layer chromatography, carried out on 0.2 mm silica gel 60 F_254_ (Merck) plates using UV light (254 and 366 nm) for detection. Common reagents grade chemicals are either commercially available and were used without further purification or prepared by standard literature procedures.

### Synthesis of benzylamide derivatives 2(a–l)

The mixture of benzyl amine (0.268 g, 2.5 mmol), acid chloride (3.5 mmol) and triethylamine (0.695 ml, 5 mmol) in DMF (5 ml) was stirred at room temperature for 5–6 h (TLC Check, 40 % ethyl acetate/hexane). To the stirring solution KF (88 mg, 1.5 mmol) was added and the reaction mixture was stirred for 0.5 h. Sucrose (0.514 g, 1.5 mmol) was added and stirring was continued for next half an hour. Water (50 ml) was then added to the reaction mixture under stirring. The products 2a–e and 2g–l were isolated by filtration and product 2f was extracted in EtOAc (25 ml).

Cyclopentanecarboxylic acid benzylamide 2a: white crystalline solid; [M.p. 94 °C, *lit. mp 94* °C (Sudrik et al. 2002); yield 498 mg, 98 %] Analysis: IR (KBr): 3354, 1654, 1601 cm^−1^; ^1^H NMR (300 MHz, DMSO-*d*_*6*_) δ 8.26 (bs, 1H, NH), 7.19–7.33 (m, 5H, ArH), 4.24 (d, J = 6.0 Hz, 2H, CH_2_), 2.52–2.72 (m, 1H, CH), 1.45–1.80 (m, 8H, 4 × CH_2_).

N-Benzyl-2, 2-dimethyl-propionamide 2b: white crystalline solid; [M.p. 83 °C, *lit. Mp 81*–*84* °C (Martínez-Asencio et al. 2011a), yield 474 mg, 99 %] Analysis: IR (KBr): 3358, 1660, 1610 cm^−1^; ^1^H NMR (300 MHz, DMSO-*d*_*6*_) δ 8.04 (t, J = 5.5 Hz, 1H, NH), 7.17–7.33 (m, 5H, ArH), 4.25 (d, J = 6.0 Hz, 2H, CH_2_), 1.12 (s, 9H, 3 × CH_3_).

N-Benzyl-4-fluoro-benzamide 2c: white crystalline solid [M.p. 144 °C, *lit. mp 143*–*144* °C (Mamat et al. 2011) yield 567 mg, 99 %] Analysis: IR (KBr): 3365, 1647, 1611 cm^−1^; ^1^H NMR (300 MHz, DMSO-*d*_*6*_) δ 9.07 (t, J = 5.9 Hz, 1H, NH), 7.93–8.01 (m, 2H, ArH), 7.22–7.36 (m, 7H, ArH), 4.48 (d, J = 6.0 Hz, 2H, CH_2_). ^13^C NMR (300 MHz, DMSO-*d*_*6*_) δ 43.1, 115.5, 115.8, 127.2, 127.7, 128.7, 130.3, 130.4, 131.2, 131.3, 140.0, 162.7, 165.6.

Cyclopropanecarboxylic acid benzylamide 2d: white crystalline solid [M.p. 140 °C, *lit.mp 140*–*141* °C (Yang and Shi 2005) yield 412 mg, 94 %] Analysis: IR (KBr): 3370, 1665, 1605 cm^−1^; ^1^H NMR (300 MHz, DMSO-*d*_*6*_) δ 8.54 (br. s., 1H, NH), 7.20–7.35 (m, 5H, ArH), 4.27 (d, J = 6.0 Hz, 2H, CH_2_), 1.55–1.64 (m, 1H, CH), 0.62–0.72 (m, 4H, 2 × CH_2_).

Cyclohexanecarboxylic acid benzylamide 2e: white crystalline solid [M.p. 108 °C, *lit. mp 107*–*109* °C (Saito et al. 2008) yield 532 mg, 98 %] Analysis: IR (KBr): 3345, 1651, 1612 cm^−1^; ^1^H NMR (300 MHz, DMSO-*d*_*6*_) δ 8.20 (br. s., 1H, NH), 7.18–7.33 (m, 5H, ArH), 4.23 (d, J = 5.7 Hz, 2H, CH_2_), 2.15 (s, 1H, CH), 1.57–1.75 (m, 5H, 5xCH), 1.10–1.42 (m, 5H, 5 × CH).

Pentanoic acid benzylamide 2f: white crystalline solid [M.p. 42 °C, *lit.mp 41.1*–*41.8* °C (Dermer and King 1943); yield 464 mg, 97 %] Analysis: IR (KBr): 3352, 1654, 1603 cm^−1^; ^1^H NMR (300 MHz, DMSO-*d*_*6*_) δ 8.28 (t, J = 5.5 Hz, 1H, NH), 7.19–7.33 (m, 5H, ArH), 4.24 (d, J = 6.0 Hz, 2H, CH_2_), 2.13 (t, J = 7.4 Hz, 2H, CH_2_), 1.41–1.55 (m, 2H, CH_2_), 1.26 (dq, J = 14.9,7.4 Hz, 2H, CH_2_), 0.78–0.89 (m, 3H, CH_3_).

Furan-2-carboxylic acid benzylamide 2g: white crystalline solid [M.p. 113 °C, *lit. mp 111*–*113* °C (Gernigon et al. 2012); yield 498 mg, 99 %] Analysis: IR (KBr): 3370, 1657, 1604 cm^−1^; ^1^H NMR (300 MHz, DMSO-*d*_*6*_) δ 8.92 (t, J = 5.9 Hz, 1H, NH), 7.77–7.85 (m, 1H, ArH), 7.20–7.35 (m, 5H, ArH), 7.13 (d, J = 3.4 Hz, 1H, ArH), 6.62 (dd, J = 3.4, 1.9 Hz, 1H, ArH), 4.42 (d, J = 6.0 Hz, 2H, CH_2_).

N-Benzyl-4-nitro-benzamide 2h: light yellow crystalline solid [M.p. 140 °C, *lit. mp 139* °C (Bahrami et al. 2010); yield 590 mg, 92 %] Analysis: IR (KBr): 3365, 1667, 1617, 1545 cm^−1^; ^1^H NMR (300 MHz, DMSO-*d*_*6*_) δ 9.39 (t, J = 6.0 Hz, 1H, NH), 8.31–8.36 (m, 2H, ArH), 8.10–8.15 (m, 2H, ArH), 7.23–7.37 (m, 5H, ArH), 4.52 (d, J = 6.0 Hz, 2H, CH_2_).

N-Benzyl-4-methoxy-benzamide 2i: white crystalline solid [M.p. 129 °C, *lit. mp125* °C (Kokare et al. 2007), yield 597 mg, 99 %] Analysis: IR (KBr): 3345, 1649, 1601 cm^−1^; ^1^H NMR (300 MHz, DMSO-*d*_*6*_) δ 8.89 (t, J = 6.0 Hz, 1H, NH), 7.84–7.91 (m, 2H, ArH), 7.20–7.34 (m, 5H, ArH), 6.97–7.04 (m, 2H, ArH), 4.46 (d, J = 6.0 Hz, 2H, CH_2_), 3.8 (s, 3H, OCH_3_).

N-Benzyl-2, 4-Dichloro-benzamide 2j: white crystalline solid [M.p. 125 °C, *lit. mp 125* °C (Richard Cremlyna et al. 1989); yield 666 mg, 95 %] Analysis: IR (KBr): 3355, 1654, 1619 cm^−1^; ^1^H NMR (300 MHz, DMSO-*d*_*6*_) δ 9.02 (s, 1H, NH), 7.69 (s, 1H, ArH), 7.49 (s, 2H, ArH), 7.22–7.37 (m, 5H, ArH), 4.44 (d, J = 6.0 Hz, 2H, CH_2_).

N-Benzyl-propionamide 2 k: white crystalline solid [M.p. 51 °C, *lit. mp 49*–*51* °C (Lee et al. 1999); yield 396 mg, 97 %] Analysis: IR (KBr): 3349, 1657, 1608 cm^−1^; ^1^H NMR (300 MHz, DMSO-*d*_*6*_) δ 8.25 (br. s., 1H, NH), 7.19–7.34 (m, 5H, ArH), 4.24 (d, J = 6.0 Hz, 2H, CH_2_), 2.13 (q, J = 7.6 Hz, 2H, CH_2_), 0.97–1.15 (m, 3H, CH_3_).

N-Benzyl-benzamide 2l: white crystalline solid [M.p. 104 °C, *lit. mp 104*–*106* °C (Nowrouzi and Jonaghani 2012); yield 523 mg, 99 %] Analysis: IR (KBr): 3358, 1656, 1610 cm^−1^; ^1^H NMR (300 MHz, DMSO-*d*_*6*_) δ 9.04 (t, J = 5.9 Hz, 1H, NH), 7.87–7.92 (m, 2H, ArH), 7.43–7.56 (m, 3H, ArH), 7.20–7.35 (m, 5H, ArH), 4.48 (d, J = 6.0 Hz, 2H, CH_2_). Calculated for C_14_H_13_NO (211.27): C, 79.59; H, 6.20; N, 6.63. Found: C, 79.57; H, 6.21; N, 6.62.

### Synthesis of sulfonamides derivatives 3(a–h)

The mixture of benzylamine (0.268 g, 2.5 mmol), sulfonyl chloride (3.5 mmol) and triethylamine (0.695 ml, 5 mmol) in DMF (5 ml) was stirred at room temperature for 5–6 h (TLC Check, 40 % ethyl acetate/hexane). To the stirring solution KF (88 mg, 1.5 mmol) was added and the reaction mixture was stirred for 0.5 h. Sucrose (0.514 g, 1.5 mmol) was added and stirring was continued for next half an hour. Water (50 ml) was then added to the reaction mixture under stirring. The product was isolated by filtration and washed with 10 ml water.

N-Benzyl-benzenesulfonamide 3a: white crystalline solid [M.p. 90 °C, *lit. mp 89*–*90* °C (José et al. 2011); yield 594 mg, 96 %] Analysis: IR (KBr): 3365, 1601, 1340 cm^−1^; ^1^H NMR (300 MHz, DMSO-*d*_*6*_) δ 9.04 (bs. s 1H, NH), 7.87–7.92 (m, 2H, ArH), 7.43–7.56 (m, 3H, ArH), 7.20–7.35 (m, 5H, ArH), 4.48 (d, J = 6.0 Hz, 2H, CH_2_).

N-Benzyl-4-methyl-benzenesulfonamide 3b: white crystalline solid [M.p. 111 °C, *lit. mp 111* °C (Gao et al. 2005); yield 647 mg, 99 %] Analysis: IR (KBr): 3361, 1615, 1338 cm^−1^; ^1^H NMR (300 MHz, DMSO-*d*_*6*_) δ 8.22 (bs, 1H, NH), 7.69–7.94 (m, 2H, ArH), 7.33–7.52 (m, 2H, ArH), 7.19–7.32 (m, 5H, ArH), 4.00 (s, 2H, CH_2_), 1.90 (s, 3H, CH_3_).

N-Benzyl-4-fluoro-benzenesulfonamide 3c: white crystalline solid [M.p. 99 °C, *lit. mp 96*–*98* °C (Ramunno et al. 2012), yield 644 mg, 97 %] Analysis: IR (KBr): 3355, 1611, 1338 cm^−1^; ^1^H NMR (300 MHz, DMSO-*d*_*6*_) δ 8.22 (bs, 1H, NH), 7.69–7.94(m, 2H, ArH), 7.33–7.52 (m, 2H, ArH), 7.19–7.32 (m, 5H, ArH), 4.00 (s, 2H, CH_2_). ^13^C NMR (300 MHz, DMSO-*d*_*6*_) δ: 46.6, 116.5, 116.8, 127.6, 128.1, 128.7, 129.9, 130.0, 137.6, 137.7, 137.9, 162.8, 166.1.

N-Benzyl-4-chloro-benzenesulfonamide 3d: white crystalline solid [M.p. 105 °C, *lit. mp 104*–*106* °C (Ramunno et al. 2012) yield 690 mg, 98 %] Analysis: IR (KBr): 3365, 1614, 1335 cm^−1^; ^1^H NMR (300 MHz, DMSO-*d*_*6*_) δ 8.25 (bs, 1H, NH), 7.75–7.98 (m, 2H, ArH), 7.38–7.54 (m, 2H, ArH), 7.19–7.32 (m, 5H, ArH), 4.02 (s, 2H, CH_2_).

N-Benzyl-4-trifluromethoxy-benzenesulfonamide 3e: white crystalline solid [M.p. 114 °C, *lit. mp 114* °C (Shi et al. 2009); yield 779 mg, 94 %] Analysis: IR (KBr): 3348, 1603, 1341 cm^−1^; ^1^H NMR (300 MHz, DMSO-*d*_*6*_) δ 8.44 (bs, 1H, NH), 7.76–8.01 (m, 4H, ArH), 7.11–7.28 (m, 5H, ArH), 4.04 (s, 2H, CH_2_).

N-Benzyl-4-methoxy-benzenesulfonamide 3f: white crystalline solid [M.p. 113 °C, *lit. mp 112*–*113* °C (Martínez-Asencio et al. 2011b); yield 686 mg, 99 %] Analysis: IR (KBr): 3357, 1607, 1344 cm^−1^; ^1^H NMR (300 MHz, DMSO-*d*_*6*_) δ 8.42 (bs, 1H, NH), 7.84–7.91 (m, 2H, ArH), 7.20–7.34 (m, 5H, ArH), 6.97–7.04 (m, 2H, ArH), 4.46 (d, J = 6.0 Hz, 2H, CH_2_), 3.95 (s, 3H, OCH_3_).

N-Benzyl-2, 4-dichloro-benzenesulfonamide 3g: white crystalline solid [M.p. 115 °C, *yield* 775 mg, 98 %] Analysis: Calculated for C_13_H_11_Cl_2_NO_2_S (316.21): C, 49.38; H, 3.51; N, 4.43. Found: C, 49.37; H, 3.52; N, 4.42. IR (KBr): 3358, 1605, 1342 cm^−1^; ^1^H NMR (300 MHz, DMSO-*d*_*6*_) δ 8.56 (s, 1H, NH), 7.86 (d, J = 8.7 Hz, 1H, ArH), 7.67–7.76 (m, 1H, ArH), 7.44–7.60 (m, 1H, ArH), 7.12–7.26 (m, 5H, ArH), 4.09 (s, 2H, CH_2_).

N-Benzyl-3, 4-dichloro-benzenesulfonamide 3h: white crystalline solid [M.p. 112 °C; yield 767 mg, 97 %] Analysis: Calculated for C_13_H_11_Cl_2_NO_2_S (316.21): C, 49.38; H, 3.51; N, 4.43. Found: 49.40; H, 3.52; N, 4.42. IR (KBr): 3354, 1605, 1343 cm^−1^; ^1^H NMR (300 MHz, DMSO-*d*_*6*_) δ 8.40 (bs, 1H, NH), 7.86 (d, J = 1.9 Hz, 1H, ArH), 7.80 (d, J = 8.7 Hz, 1H, ArH), 7.65–7.75 (m, 1H, ArH), 7.13–7.29 (m, 5H, ArH), 4.05 (s, 2H, CH_2_). ^13^C NMR (300 MHz, DMSO-*d*_*6*_) δ: 46.7, 127.1, 127.7, 128.2, 128.6, 128.8, 131.9, 132.4, 135.7, 137.5, 141.7.

### Synthesis of urea derivatives 4(a–i)

The mixture of benzylamine (0.268 g, 2.5 mmol), isocyanate (3.5 mmol) and triethylamine (0.695 ml, 5 mmol) in DMF (5 ml) was stirred at room temperature for 5–6 h (TLC Check, 50 % ethyl acetate/hexane). To the above stirring solution, KF (88 mg, 1.5 mmol) was added and the reaction mixture was stirred for 0.5 h. Sucrose (0.514 g, 1.5 mmol) was added and stirring was continued for next half an hour. Water (50 ml) was then added to the reaction mixture under stirring. The product was isolated by filtration and washed with 10 ml water.

1-Benzyl -3-(3-cyano-phenyl)-urea 4a: white crystalline solid [M.p. 176 °C; yield 616 mg, 98 %] Analysis: IR (KBr): 3454, 2202, 1640, 1602 cm^−1^; ^1^H NMR (300 MHz, DMSO-*d*_*6*_) δ 8.94 (s, 1H, NH), 7.95 (t, J = 1.7 Hz,1H, NH), 7.60 (ddd, J = 8.1, 2.3, 1.3 Hz, 1H, ArH), 7.43 (t, J = 7.9 Hz, 1H, ArH), 7.19–7.39 (m, 6H, ArH), 6.81 (t, J = 5.9 Hz, 1H, ArH), 4.31 (d, J = 6.0 Hz, 2H, CH_2_). ^13^C NMR (300 MHz, DMSO-*d*_*6*_) δ: 43.2, 111.9, 119.4, 120.6, 122.7, 125.0, 127.2, 127.6, 128.8, 130.5, 140.5, 141.8, 155.4.

1-Benzyl-3-naphthalen-1-yl-urea 4b: white crystalline solid [M.p. 203 °C; yield 684 mg, 99 %] Analysis: Calculated for C_18_H_16_N_2_O (276.34): C, 78.24; H, 5.84; N, 10.14. Found: C, 78.22; H, 5.85; N, 10.13. IR (KBr): 3374, 1638, 1605 cm^−1^; ^1^H NMR (300 MHz, DMSO-*d*_*6*_) δ 8.64 (s, 1H, NH), 8.11 (d, J = 7.6 Hz, 1H, ArH), 8.05 (dd, J = 7.7, 0.9 Hz, 1H, ArH), 7.85–7.98 (m, 1H, ArH), 7.66 (s, 1H, ArH), 7.24–7.59 (m, 10H, ArH), 7.06 (t, J = 5.9 Hz, 1H, ArH), 4.39 (d, J = 5.7 Hz, 2H, CH_2_). ^13^C NMR (300 MHz, DMSO-*d*_*6*_) δ: 46.7, 127.1, 127.7, 128.2, 128.6, 128.8, 131.9, 132.4, 135.7, 137.5, 141.7.

1-Benzyl-3-(4-fluoro-phenyl)-urea 4c: white crystalline solid [M.p. 179 °C; yield 592 mg, 97 %] Analysis: Calculated for C_14_H_13_FN_2_O (244.27): C, 68.84; H, 5.36; N, 11.47. Found: C, 68.83; H, 5.35; N, 11.46. IR (KBr): 3354, 1635, 1602 cm^−1^; ^1^H NMR (300 MHz, DMSO-*d*_*6*_) δ 8.87 (s, 1H, NH), 8.2 (t, J = 5.9 Hz, 1H, NH), 7.93–8.01 (m, 2H, ArH), 7.15–7.25 (m, 5H, ArH), 7.25–7.36 (m, 2H, ArH), 4.48 (d, J = 6.0 Hz, 2H, CH_2_).

1-Benzyl-3-(2-trifluoromethyl-phenyl)-urea 4d: white crystalline solid [M.p. 168 °C; yield 721 mg, 98 %] Analysis: Calculated for C_15_H_13_F_3_N_2_O (294.28): C, 61.22; H, 4.45; N, 9.52. Found: C, 61.20; H, 4.44; N, 9.53. IR (KBr): 3370, 1645, 1601 cm^−1^; ^1^H NMR (300 MHz, DMSO-*d*_*6*_) δ 8.72 (s, 1H, NH), 7.60–7.97 (m, 4H, ArH), 7.45 (t, J = 5.7 Hz, 1H, NH), 7.25–7.35 (m, 5H, ArH), 4.31 (d, J = 5.7 Hz, 2H).

1-Benzyl-3-(2-methoxy- phenyl)-urea 4e: white crystalline solid [M.p. 177 °C; yield 622 mg, 97 %] Analysis: Calculated for C_15_H_16_N_2_O_2_ (256.31): C, 70.29; H, 6.29; N, 10.93. Found: C, 70.31; H, 6.28; N, 10.94. IR (KBr): 3365, 1636, 1607 cm^−1^; ^1^H NMR (300 MHz, DMSO-*d*_*6*_) δ 8.72 (s, 1H, NH),7.08–7.36 (m, 7H, ArH), 6.85–6.95 (m, 1H, ArH), 6.69 (bs, 1H, NH), 6.44–6.58 (m,1H, ArH), 4.29 (bs, 2H, CH_2_), 3.5 (s, 3H, OCH_3_).

1-Benzyl-3-phenyl-urea 4f: white crystalline solid [M.p. 194 °C; yield 560 mg, 99 %] Analysis: Calculated for C_14_H_14_N_2_O (226.28): C, 74.31; H, 6.24; N, 12.38. Found: C, 74.33; H, 6.25; N, 12.39. IR (KBr): 3364, 1643, 1602 cm^−1^; ^1^H NMR (300 MHz, DMSO-*d*_*6*_) δ 8.60 (s, 1H, NH), 7.08–7.40 (m, 8H, ArH), 6.879 (ddd, J = 8.0, 2.0, 0.9 Hz, 1H, ArH), 6.63 (t, J = 6.0 Hz, 1H, NH), 6.47 (ddd, J = 8.3, 2.6, 0.9 Hz, 1H, ArH), 4.33(d, J = 6.0 Hz, 2H, CH_2_).

1-Benzyl-3-(4-cyano-phenyl)-urea 4g: white crystalline solid [M.p. 198 °C; yield 578 mg, 92 %] Analysis: Calculated for C_15_H_13_N_3_O (251.29): C, 71.70; H, 5.21; N, 16.72. Found: C, 71.73; H, 5.22; N, 16.71. IR (KBr): 3374, 2201, 1643, 1608 cm^−1^; ^1^H NMR (300 MHz, DMSO-d6) δ 8.80 (s, 1H, NH), 7.39–7.62(m, 1H, ArH), 7.19–7.36 (m, 5H, ArH), 7.32 (bs, 1H, NH), 6.96–7.11 (m, 1H, ArH), 6.62–6.78 (m, 2H), 4.29 (d, J = 6.0 Hz, 2H, CH2).

1-Benzyl-3-(4-trifluoromethoxy-phenyl) urea 4h: white crystalline solid [M.p. 192 °C; yield 730 mg, 94 %] Analysis: Calculated for C_15_H_13_F_3_N_2_O_2_ (310.28): C, 58.07; H, 4.22; N, 18.37. Found: C, 58.06; H, 4.21; N, 18.36. IR (KBr): 3374, 1644, 1610 cm^−1^; ^1^H NMR (300 MHz, DMSO-*d*_*6*_) δ 8.80 (s, 1H, NH), 7.5 (d, J = 7.6 Hz, 2H, ArH), 7.18–7.22 (m, 2H, ArH), 7.22–7.36 (m, 5H, ArH), 6.69 (t, J = 6.0 Hz, 1H, NH), 4.30 (d, J = 6.0 Hz, 2H, CH_2_).

1-Benzyl-3-(2,5-Dimethoxy-phenyl) urea 4i: white crystalline solid [M.p. 179 °C; yield 709 mg, 99 %] Analysis: Calculated for C_16_H_18_N_2_O_3_ (286.33): C, 67.12; H, 6.34; N, 9.78. Found: C, 67.11; H, 6.33; N, 9.77. IR (KBr): 3374, 1638, 1601 cm^−1^; ^1^H NMR (300 MHz, DMSO-*d*_*6*_) δ 8.03 (s, 1H, NH), 7.8–7.84 (m, 1H, ArH), 7.21–7.38 (m, 6H, ArH & NH), 6.83–6.91 (m, 1H, ArH), 6.38–6.50 (m, 1H, ArH), 4.28 (d, J = 5.7 Hz, 2H), 3.75 (s, 3H, OCH_3_), 3.65 (s, 3H, OCH_3_).
